# Amlodipine-induced gingival overgrowth

**DOI:** 10.4103/0972-124X.60231

**Published:** 2009

**Authors:** M. G. Triveni, C. Rudrakshi, D. S. Mehta

**Affiliations:** *Departments of Periodontology and Implantology, Bapuji Dental College and Hospital, Davangere, Karnataka, India*

**Keywords:** Amlodipine, calcium channel blockers, gingival overgrowth

## Abstract

Gingival overgrowth represents an over-exuberant response to a variety of local and systemic conditions. Certain anticonvulsants, immuno-suppressive drugs and a number of calcium channel blockers have been shown to produce similar gingival overgrowths in certain susceptible patients. Amlodipine is a comparatively new calcium channel blocker and has been used with increasing frequency in the management of hypertension and angina. Although amlodipine is considered as a safe drug, very rarely it may induce gingival overgrowth also. A rare case of amlodipine-induced gingival overgrowth has been reported herein in a 50-year-old female patient. The treatment aspect included Phase-1 therapy, substitution of the drug, the surgical excision and the maintenance and supportive therapy resulting in excellent clinical outcome.

## INTRODUCTION

Gingival overgrowth is one of the most important clinical features of gingival pathology. It has multifactorial etiologies and has been frequently associated with inflammatory changes in the gingiva. Other factors related to this condition are hereditary (familial), malignancies and those resulting from adverse effects associated with systemic administration of certain drugs.[1]

Drug-induced gingival overgrowth (DIGO) remains a significant problem for the dental clinicians and the periodontologists. Patients medicated with certain drugs may be implicated in this unwanted side effect (DIGO), which may interfere with esthetics, mastication or speech. Disfiguring gingival overgrowth triggered by these medications is not only esthetically displeasing but also often impairing nutrition and access for oral hygiene resulting in an increased susceptibility to oral infection, caries and periodontal diseases.[2]

An increasing number of medications are associated with gingival overgrowth. Currently, more than 20 prescription medications are associated with gingival enlargement.[3] Drugs associated with gingival overgrowth can be broadly divided into three categories: Anticonvulsants, calcium channel blockers, and immunosuppressants. Although the pharmacologic effect of each of these drugs is different and directed toward various primary target tissues, all of them seem to act similarly on a secondary target tissue, that is, the gingival connective tissue causing common clinical and histopathological findings.

Amlodipine is a new dihydropyridine calcium channel blocker that is used in the management of both hypertension and angina. Ellis *et al*.,[4] first reported gingival sequestration of amlodipine and amlodipine-induced gingival overgrowth. Since then, very few isolated cases of AIGO have appeared in the dental literature although there are numerous reports of nifedipine (another member of calcium channel blockers)-induced gingival overgrowth till date.

Clinical manifestations of gingival enlargement frequently appear within one to three months, after initiation of treatment with the associated medications. Gingival overgrowth normally begins at the interdental papillae and is more frequently found in the anterior segment of the labial surfaces. Gradually, gingival lobulations are formed that may appear inflamed or fibrotic in nature depending on the degree of local factor-induced inflammation. However, the fibrotic enlargement is normally confined to the attached gingiva, but may extend coronally causing the extensive disfigurement of gingiva.

In the present report, a case of amlodipine-induced gingival overgrowth has been presented wherein the AIGO was treated in the following phases: (1) thorough Phase-1 therapy, (2) substitution of the drug, (3) surgical excision of the residual gingival overgrowth and (4) maintenance and supportive therapy.

## CASE REPORT

A 50-year-old female patient came to the Department of Periodontics, Bapuji Dental College and Hospital, Davangere with the chief complaint of enlarged gums in the upper and lower front tooth region for three months. Patient was not aware of such growth until three months back when she noticed a small bead-like nodular enlargement of the gums that gradually progressed to the present size covering almost the entire front teeth. Enlargement was not associated with bleeding from the gums and loosening of teeth. Her past medical history revealed that the patient was hypertensive for last four years and was under medication (Amlodipine 5 mg, once daily). However, her past dental history was noncontributory.

Her personal history reveled that she was a regular betel nut chewer. She used to clean her teeth once daily with brush and paste, which she discontinued recently because of the coverage of teeth with the enlarged gums. Her general physical examination revealed that the patient was moderately built and her vital signs were within the normal range. There were no significant extraoral findings.

**Figure 1:**
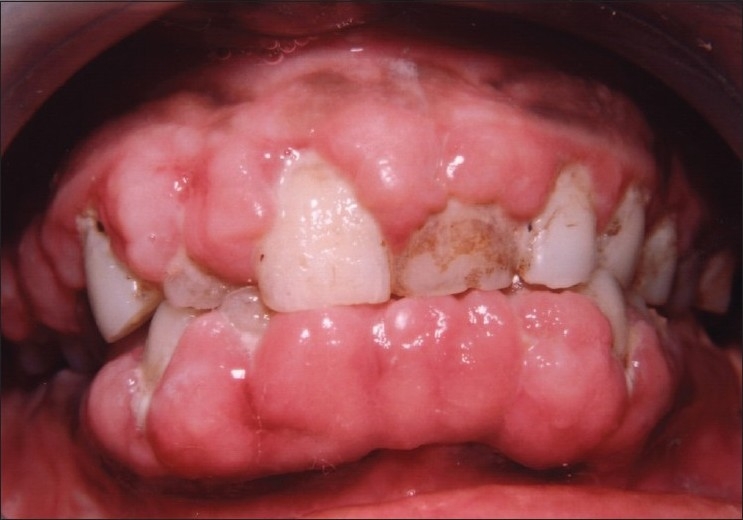
Preoperative view showing extensive gingival overgrowth (facial view)

**Figure 2:**
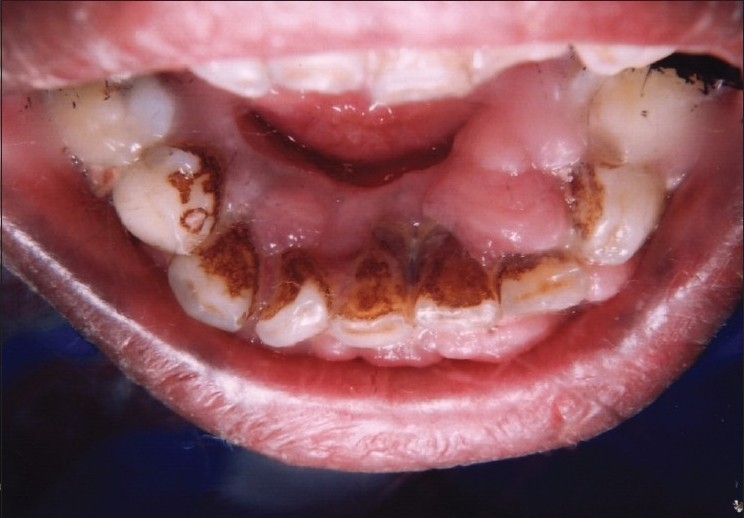
Preoperative view showing gingival overgrowth in lingual side of lower anteriors (lingual view)

On intraoral examination, marginal and interdental gingival enlargement was well appreciated covering almost coronal one-third of maxillary and mandibular anterior teeth [Figures 1 and 2]. Gingiva was pink in colour with erythematous area and lobulated surface. Margins of the gingiva were rolled out with loss of normal gingival scalloping. On palpation, gingiva was firm and resilient in consistency. Hypertrophied areas were painless and did not bleed on touch. Poor oral hygiene status of the patient was assessed from the presence of local irritating factors contributing to the mild inflammatory component of the gingival enlargement. The probing of gingival sulcus revealed presence of pseudo-pockets and elicited the bleeding.

Patient was subjected to complete hemogram and all the parameters were found to be within normal range. Orthopantomogram revealed complete set of dentition with generalized bone loss. Histopathological report revealed mixture of dense and loose fibrous component with inflammatory cell infiltrate and few areas of calcifications in the stroma. On the basis of the patient's history and clinical features, a clinical diagnosis of amlodipine induced gingival overgrowth (AIGO) was made.

The treatment of the patient was started with nonsurgical approach. Patient was subjected to Phase-1 therapy including the planned sessions of scaling and root planing. Patient's physician was consulted regarding drug substitution or withdrawal of the drug. The physician substituted the drug with tab. Normadate 100 mg (Labetolol). Patient was instructed to maintain good oral hygiene with the use of chlorhexidine oral rinses.

**Figure 3:**
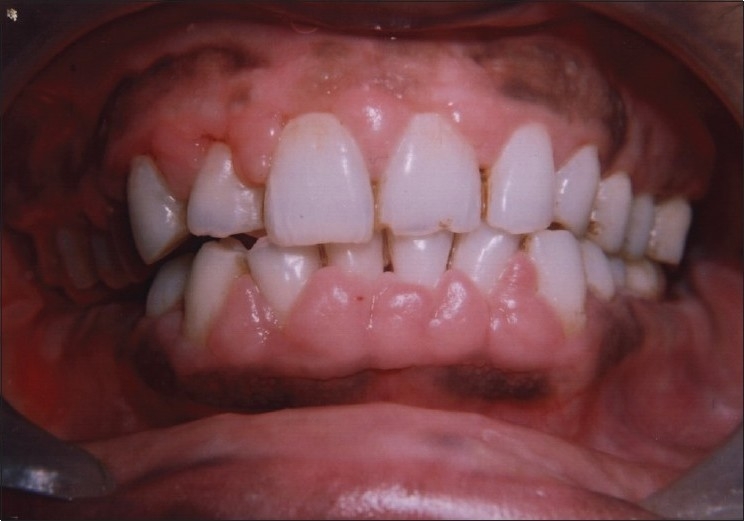
Three months after change of drug

**Figure 4:**
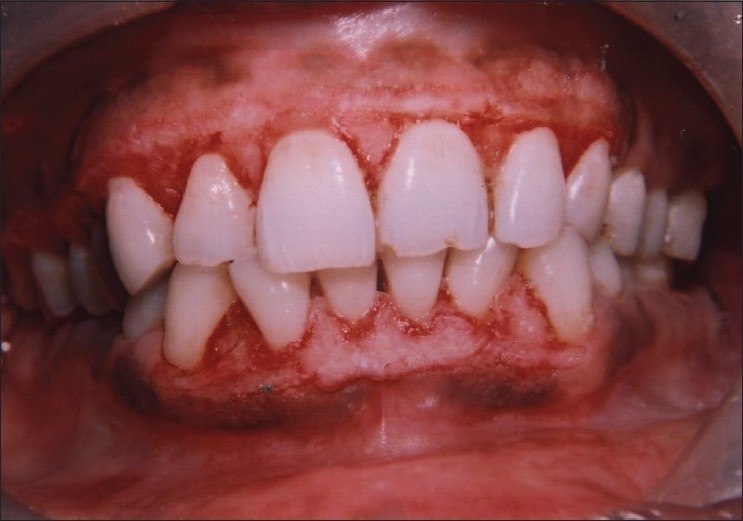
Immediate postoperative view after gingivectomy and gingivoplast

**Figure 5:**
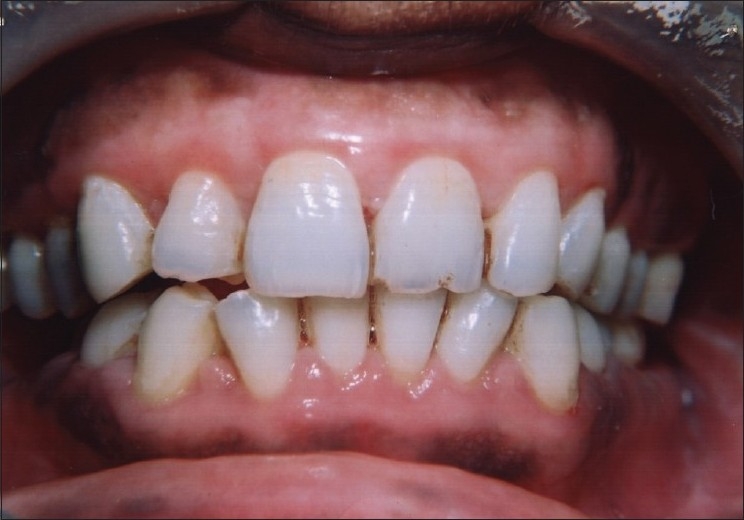
Three months postsurgically (facial view)

**Figure 6:**
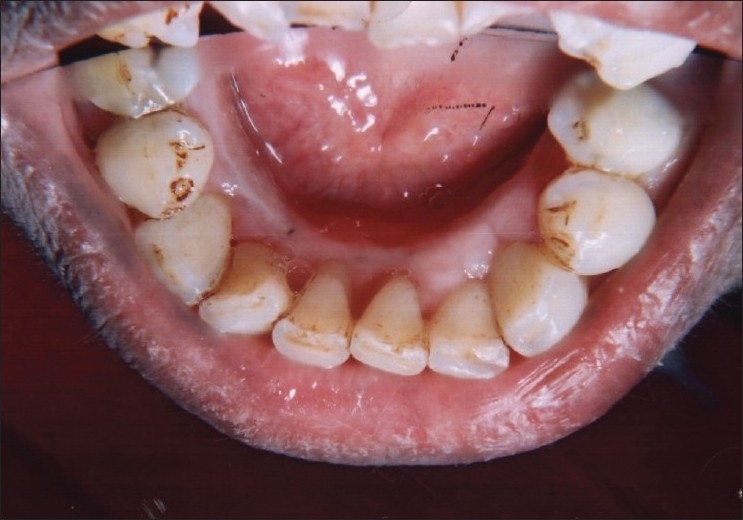
Three months postsurgically (lingual view)

A dramatic response was noticed after three months of drug substitution and maintenance of regular oral hygiene. There was regression in the size of gingival enlargement with minimal of fibrotic component left [Figure 3]. Finally, surgical excision of gingival hyperplastic tissue was planned employing the techniques of gingivectomy/gingivoplasty to restore the normal shape and contour of the gingiva [Figure 4]. Postoperatively, there was successful elimination of enlarged gingival tissue and restoration of a physiological gingival contour giving the patient an esthetically pleasing appearance [Figures 5 and 6].

## DISCUSSION

‘Gingival overgrowth’ or ‘gingival enlargement’ is the preferred term for many of these medication-related gingival conditions previously labeled as *gingival hyperplasia* or *gingival hypertrophy*. These earlier terms do not accurately reflect our current understanding of the macroscopically enlarged and histologically altered gingiva. Drugs associated with gingival overgrowth can be broadly categorized into three major groups according to their therapeutic actions, namely, anticonvulsants, immunosuppressants and calcium channel blockers.[3]

The widespread use of calcium channel blockers began in 1980s. The dihydropyridones (e.g. nifedipine) tend to be more commonly associated with the gingival enlargement than with other sub groups of calcium channel antagonists such as amlodipine. Prescription of calcium channel blockers is relatively common, making it difficult to determine the true incidence of drug-induced gingival enlargement.

Amlodipine is a third generation dihydropyridine calcium antagonist, which has a mode of action pharmacodynamically comparable to nifedipine.[5] However, amlodipine has a unique physiochemical profile, which is characterized by near complete absorption, late-peak plasma concentrations, high bioavailability and slow hepatic biodegradation. The associated slow elimination of amlodipine with resulting long duration of its action means that only a single-daily dose is required. This in turn results in better patient compliance and has until now been associated with similar or reduced severity of side effects compared with nifedipine.[5,6]

Patients taking nifedipine appear to be at increased risk for developing significant overgrowth than those on amlodipine. The difference between nifedipine and amlodipine is of interest, as both drugs are dihydropyridones and hence structurally similar. Also, both are secreted in the gingival crevicular fluid, but differ in their physico-chemical profile. Amlodipine is more polar than the other dihydropyridones, with a p*K*a value of 8.7. In contrast, nifedipine is intensely lipophilic and will readily dissolve within the cell membrane and pass into the cytoplasm. While the mechanism of drug-induced gingival overgrowth is considered to be multifactorial, the drug/cellular interaction is pivotal in the pathogenesis of this effect.[7]

The clinician should emphasize plaque control as the first step in the treatment of drug-induced gingival enlargement. Although the exact role played by bacterial plaque in drug-induced gingival enlargement is unclear, there is evidence that elimination of local factors and regular maintenance of good oral hygiene decrease the degree and severity of the gingival enlargement and improve the overall gingival health. Usually, a three-month interval for periodontal maintenance therapy has been recommended in DIGO.

The treatment options for drug-induced gingival enlargement should be based on the medication being used and the clinical presentation of the individual case. First, consideration should be given to the possibility of discontinuing or substituting the drug. Either of those scenarios should be examined in consultation with the patient's physician. Simple discontinuation of the offending agent is usually not a practical solution. However, its replacement with another medication might be the practical solution. It may take from 1 to 8 weeks for resolution of gingival overgrowth. Consideration may be given to the use of another class of antihypertensive medications, which are known to be not-associated with the gingival enlargement. In the present case, substitute drug, that is, Normadate 100 mg along with Phase-1 therapy resulted in clinically significant improvement in six weeks time.

The need for, and timing of, any surgical intervention needs to be carefully assessed. Surgery is normally performed for cosmetic/aesthetic needs before any functional consequences are present. The classical surgical approach has been the external bevel gingivectomy. However, a total or partial internal gingivectomy approach has been suggested as an alternative. In the present report, as the gingival overgrowth was not associated with the true periodontal pockets and the osseous defects, external bevel gingivectomy followed by gingivoplasty was carried out. The postoperative results were found to be extremely satisfactory both esthetically and functionally.
